# Direct N-Glycosylation Profiling of Urine and Prostatic Fluid Glycoproteins and Extracellular Vesicles

**DOI:** 10.3389/fchem.2021.734280

**Published:** 2021-09-27

**Authors:** Calvin R. K. Blaschke, Jordan P. Hartig, Grace Grimsley, Liping Liu, O. John Semmes, Jennifer D. Wu, Joseph E. Ippolito, Chanita Hughes-Halbert, Julius O. Nyalwidhe, Richard R. Drake

**Affiliations:** ^1^ Department of Cell and Molecular Pharmacology and Experimental Therapeutics, Medical University of South Carolina, Charleston, SC, United States; ^2^ Department of Microbiology and Molecular Cell Biology, Eastern Virginia Medical School, Norfolk, VA, United States; ^3^ The Leroy T. Canoles Jr., Cancer Research Center, Eastern Virginia Medical School, Norfolk, VA, United States; ^4^ Departments of Urology and Microbiology-Immunology, Northwestern University Feinberg School of Medicine, Chicago, IL, United States; ^5^ Department of Radiology, Washington University School of Medicine, St. Louis, MO, United States; ^6^ Department of Psychiatry and Behavioral Sciences, Medical University of South Carolina, Charleston, SC, United States; ^7^ Hollings Cancer Center, Medical University of South Carolina, Charleston, SC, United States

**Keywords:** glycosylation, N-glycan, prostate, urine, MALDI

## Abstract

Expressed prostatic secretions (EPS), also called post digital rectal exam urines, are proximal fluids of the prostate that are widely used for diagnostic and prognostic assays for prostate cancer. These fluids contain an abundant number of glycoproteins and extracellular vesicles secreted by the prostate gland, and the ability to detect changes in their N-glycans composition as a reflection of disease state represents potential new biomarker candidates. Methods to characterize these N-glycan constituents directly from clinical samples in a timely manner and with minimal sample processing requirements are not currently available. In this report, an approach is described to directly profile the N-glycan constituents of EPS urine samples, prostatic fluids and urine using imaging mass spectrometry for detection. An amine reactive slide is used to immobilize glycoproteins from a few microliters of spotted samples, followed by peptide N-glycosidase digestion. Over 100 N-glycan compositions can be detected with this method, and it works with urine, urine EPS, prostatic fluids, and urine EPS-derived extracellular vesicles. A comparison of the N-glycans detected from the fluids with tissue N-glycans from prostate cancer tissues was done, indicating a subset of N-glycans present in fluids derived from the gland lumens. The developed N-glycan profiling is amenable to analysis of larger clinical cohorts and adaptable to other biofluids.

## Introduction

In the search and characterization of disease biomarkers for use in liquid biopsy applications, proximal fluids like blood and urine are commonly used. Proximal fluids are found adjacent to a given tissue or organ and represent a repertoire of secreted proteins and shed cells reflective of the physiological state of that tissue. For prostate cancer and other genitourinary diseases, proximal fluids are represented by seminal plasma and expressed-prostatic secretion in urine (EPSu) (Drake and Kislinger, 2014; Nawaz et al., 2014). EPSu, also termed post-digital rectal exam (DRE) urine, represents the fluid being secreted by the prostate following a digital rectal prostate massage, which in turn can be collected in voided urine post-exam (Drake et al., 2009; Drake and Kislinger, 2014). The prostate gland secretes many proteins and other biomolecules in a prostatic fluid that combines with seminal fluid and sperm from the seminal vesicles during ejaculation. Many of these prostatic proteins are glycoproteins, like prostate specific antigen (PSA), that perform functions to activate sperm and suppress the vaginal immune micro-environment. Our group has previously characterized the proteomic composition of EPSu and prostatic secretions, identifying hundreds of different prostate-derived glycoproteins (Drake et al., 2009; Drake et al., 2010; Kim et al., 2012; Principe et al., 2012). Development and evaluation of extensive targeted proteomic assays to these proteins in EPSu are in progress for use in prostate cancer diagnosis (Kim et al., 2016; Otto et al., 2020). The prostatic fluids, EPSu and urine are also rich in extracellular vesicles (EV), which are a source for many ongoing non-coding RNA and related oligonucleotide-targeted diagnostic assays for prostate cancer and multiple diseases (Van Gils et al., 2007; Laxman et al., 2008; Linxweiler and Junker, 2020). EV obtained from urine and EPSu continues to be a highly active area for diagnostic assay development (Wang et al., 2020; Erdbrügger et al., 2021).

The majority of proteins in EPSu, urine, and associated EVs are glycosylated (Drake et al., 2009; Drake et al., 2010; Kim et al., 2012; Principe et al., 2012), either on asparagine residues, termed N-linked glycosylation, or on serine or threonine residues, termed O-glycosylation. Changes in glycosylation have been well documented in prostate cancer associated tissues, cells and biofluids (Drake et al., 2015; Scott and Munkley, 2019; Tkac et al., 2019). Extensive glycoproteomic approaches, i.e., characterization of the glycan structures at the peptide sites of modification, have been reported for urine and EPSu glycoprotein targets (Leymarie et al., 2013; Saraswat et al., 2015; Brown et al., 2020). One of the most highly characterized glycoproteins is PSA (White et al., 2009; Leymarie et al., 2013; Nyalwidhe et al., 2013; Jia et al., 2017; Kammeijer et al., 2018; Hatakeyama et al., 2021), due to its known role in prostate cancer diagnosis and relatively simple glycosylation pattern of having a single N-linked glycosylation site. Although changes in glycosylation of PSA and many other prostatic glycoproteins have diagnostic potential, assays to efficiently characterize N-glycans in urine can be lengthy and require multiple processing steps, precluding large scale clinical utility (Song et al., 2019; Hanzawa et al., 2021). Lectin arrays have been effectively used to profile glycan motifs in large cohorts of clinical urine samples (Kawakita et al., 2021; Mise et al., 2021), however this approach cannot determine full glycan compositions or distinguish N-linked or O-linked origins. Based on an adaptation of a recently published workflow for rapid characterization of serum and plasma N-glycans (Blaschke et al., 2020), we report herein a more efficient slide-based approach combined with MALDI imaging mass spectrometry (IMS) workflows to detect total N-glycan profiles of urine, EPSu and prostatic fluid samples. Comparative results with N-glycans detected and histologically mapped in prostate cancer tissues by MALDI IMS are also included.

## Materials and Methods

### Materials

Amicon Ultra 10 k centrifugal filters were obtained from Merk Millipore (Carrigtwohill, IRL). Hydrogel coated slides (Nexterion® Slide H) were obtained from Applied Microarrays (Tempe, AZ). The rotary tool was a Dremel 200 series. The well slide module (ProPlate Multi-Array Slide System, 64-well) was obtained from Grace Bio-Laboratories (Bend, OR). Sodium bicarbonate, trifluoroacetic acid (TFA), and α-cyano-4-hydroxycinnamic acid (CHCA) were obtained from Sigma-Aldrich (St. Louis, MO). HPLC grade water, 1X phosphate buffered saline (PBS), acetonitrile, citraconic anhydride, glacial acetic acid, methanol, xylene, and chloroform were obtained from Fisher Scientific (Hampton, NH). Ethanol was obtained from Decon Labs (King of Prussia, PA). Peptide-N-glycosidase F (PNGase F) PRIME^TM^ was from N-Zyme Scientifics (Doylestown, PA). H&E stains were obtained from Cancer Diagnostics (Durham, NC).

### Expressed Prostatic Secretion Urine Samples, Extracellular Vesicles, and Tissue

All samples were collected from patients and utilized after informed consent following Institutional Review Board-approved protocols at Urology of Virginia, Sentara Medical School, and the Eastern Virginia Medical School. All personal information or identifiers beyond diagnosis and lab results were not available to the laboratory investigators. EPS-urine samples were collected performing a gentle massage of the prostate gland during DRE prior to biopsy, as previously described (Drake et al., 2009). The massage consisted of three strokes on each side of the median sulcus of the prostate and the expressed fluid from the glandular network of the prostate was subsequently voided in urine. Pools (25–50 ml/sample) of EPSu were derived from 10 patients classified as having high grade, Gleason 8–10 tumors and 10 patients with low grade, Gleason 6, organ-confined prostate cancer as described previously (Nyalwidhe et al., 2013). For isolation of EPS-derived extracellular vesicles (EPSev), the two EPSu pools (45 ml) were centrifuged at 25,000 × g for 30 min, and the supernatant centrifuged at 100,000 × g for 4 h. The pelleted exosomes were washed twice with PBS and resuspended in 0.5 ml PBS, as previously described (Nyalwidhe et al., 2013). Direct EPS fluids (EPSd) were obtained under anesthesia prior to prostatectomy as previously described (Drake et al., 2010). A subset of 10 pairs of patient samples who provided both EPSu and EPSd were selected. Prior to glycomic analysis, 0.125 ml aliquots of each EPSu and EPSd sample were concentrated in a 10,000 MW filter cut-off 0.5 ml Amicon tube by centrifugation at 11,000 rpm in a Sorvall Legend Micro 21 benchtop microcentrifuge for 25 min. To each filtration tube was added 0.125 ml of 1X PBS, and centrifugation was repeated for 25 min. The remaining concentrated fluid, approximately 15–20 microliters, was removed to a separate vial. Each tube was rinsed with 20 microliters of PBS, and added to the concentrated sample vial (final volume 35–40 microliters). A de-identified prostate tumor tissue pair of Gleason grade 8 (4 + 4)/stage pT3b and patient-matched distal non-tumor tissue was obtained from the Hollings Cancer Center Tissue and Analysis Biorepository at the Medical University of South Carolina. A serial section of each tissue was H&E-stained according to a standardized protocol.

### Control Urine Standards

Commercial urine samples representing pooled samples from four healthy males and four females were purchased from Lee BioSolutions (Maryland Heights, MO). Prior to glycomic analysis, the control urine samples were filtered and rinsed as described for the EPS samples, except 4 ml starting volume was used with larger Amicon tubes.

### Expressed Prostatic Secretions Fluids and Urine Preparation for MALDI-IMS

The sample preparation and analysis of the EPS fluids (EPSu, EPSd, and EPSev) and urine samples were adapted from a workflow established for the glycomic analysis of serum and plasma (Blaschke et al., 2020). After a 30-min temperature equilibration in a moisture resistant pouch, an amine-reactive hydrogel coated slide was ground down with a rotary tool until if could fit into a Bruker MTP Slide Adapter II. A 64 well module was attached and outlined on to the back of the slide. Then the well module was unattached. Two microliters of sodium bicarbonate (100 mM, pH 8.0) was mixed with 1 microliter of the sample and briefly mixed. Within the outline of a well, 1 microliter was spotted onto the slide. EPSu, EPSd, and EPSev samples were spotted in technical triplicates, and the control urine samples were spotted in technical quadruplicates. The slide was placed in a humidity chamber, made from a culture dish with a Wypall × 60 paper towel lining the bottom and two rolled KimWipes saturated with distilled water on opposite sides, for 1 h on the benchtop to immobilize the samples to the slide. The slide was then dried in a desiccator for 15 min. The well module was reattached to the slide, matching the wells with the outlines drawn on previously. The samples were washed with Carnoy’s solution (10% glacial acetic acid, 30% chloroform, and 60% 200 proof ethanol) three times for 3 min each, and subsequently washed with HPLC-grade water once for 1 min. For the washing and rinsing steps, 50 microliters of solution was added to each well and dumped out of the well by inverting the slide. Following the water wash, the slide was dried in a desiccator for 30 min with the slide module attached. After detaching the slide module, a M5 TM-Sprayer (HTX Technologies) was used to spray a 0.1 mg/ml PNGase F PRIME solution in water on to the slide for 15 passes at 25 microliters/min, 1,200 mm/min, 45°C, and 3 mm spacing between passes with 10 psi nitrogen gas. The slide was then incubated in a preheated humidity chamber at 37°C for 2 h. A M5 TM-Sprayer was also used to apply the MALDI matrix solution (7 mg/ml of CHCA in 50% acetonitrile/0.1% TFA) on the slide for 10 passes at 100 microliters/min, 1,200 mm/min, 79°C, and 2.5 mm spacing between passes with 10 psi nitrogen gas.

### Prostate Tissue Preparation for MALDI-IMS

The tissues were prepared as described previously (Drake et al., 2018a). Briefly, the tissues were dewaxed by 1 h in 60°C and xylene washes, rehydrated with a gradation of ethanol and water washes, and underwent antigen retrieval in citraconic anhydride buffer (25-μL citraconic anhydride, 2-μL 12 M HCl, 50-ml HPLC-grade water, pH 3.0 ± 0.5) in a decloaking chamber at 95°C for 30 min. A M5 TM-Sprayer (HTX Technologies) was used to spray a 0.1 mg/ml PNGase F PRIME solution in water on to the slide for 15 passes at 25 microliters/min, 1,200 mm/min, 45°C, and 3 mm spacing between passes with 10 psi nitrogen gas. The slide was then incubated in a preheated humidity chamber at 37°C for 2 h. A M5 TM-Sprayer was also used to apply the MALDI matrix solution (7 mg/ml of CHCA in 50% acetonitrile/0.1% TFA) on the slide for 10 passes at 100 microliters/min, 1,200 mm/min, 79°C, and 2.5 mm spacing between passes with 10 psi nitrogen gas.

### MALDI Imaging Mass Spectrometry

A dual source timsTOF fleX MALDI-QTOF mass spectrometer (Bruker) was used to image the slides as previously described (McDowell et al., 2021). Images were collected with a SmartBeam 3D laser operating at 10,000 Hz with a 20 µm laser spot size at a 150 µm raster with 300 laser shots per pixel. Samples were analyzed in positive ion mode spanning a m/z range of 700–4,000.

### Data Processing and Analysis

Mass spectra were imported in to SCiLS Lab software 2021a (Bruker), normalized to total ion current, and manually peak selected for N-glycans based on theoretical mass values. SCiLS was also used for individual peak visualization and quantification. Maximum mean values for each peak were exported for each sample region. Each N-glycan measurement for each sample was subtracted by the background signal in the blank well to find the absolute intensity. To account for differences in protein concentrations that could lead to higher signal intensities and detection of more low-abundance N-glycan species, N-glycan relative intensities were calculated as the absolute intensity divided by the sum of all the absolute intensities of the N-glycans found in each of the samples being compared. Comparisons of the number of N-glycans detected in each sample is also discussed, and the presence/absence of a N-glycan in each sample is noted in Supplementary Table S1. N-glycan structures were labelled with a N-glycan class or classes depending on their putative structures. Quantifications of the N-glycan classes were calculated by summing the relative intensities of the individual N-glycans belonging to each class. N-glycan profiles were also examined by grouping each N-glycan into a group depending on the presence and/or absence of mannose, fucose, sialic acid, and sulphate and comparing the summed relative intensities of the classes. When comparing individual N-glycan intensities across samples, the multiply sodiated species of sialylated and sulfated N-glycans were added together.

## Results

**FIGURE 1:**
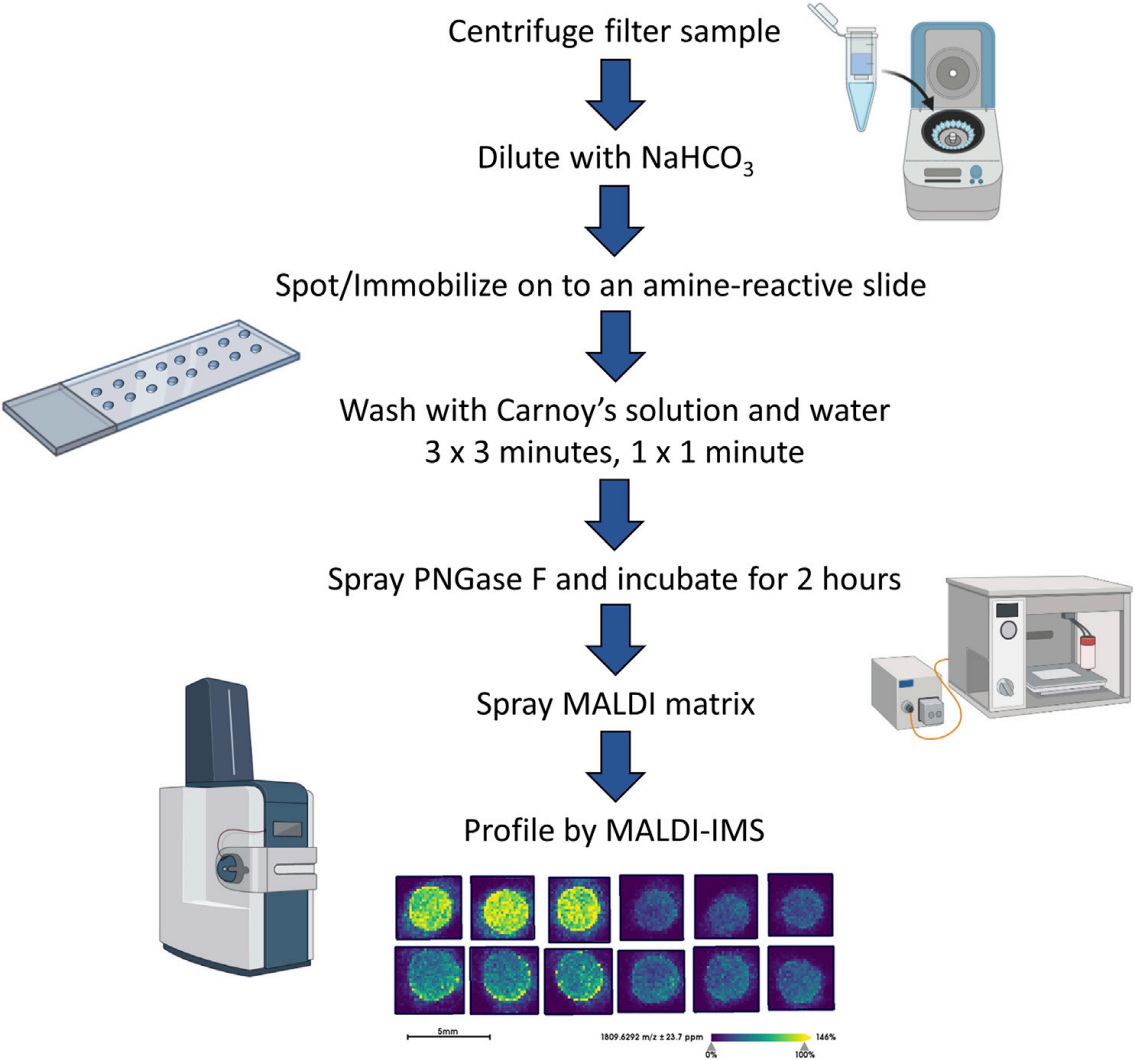
Workflow of the EPS fluid and urine N-glycan analysis.

To complement previous proteomic studies of proximal prostatic fluids obtained in the urology clinic as related to prostate cancers (Drake et al., 2009; Drake et al., 2010; Kim et al., 2012; Principe et al., 2012) a series of different EPSu, EPSd and EPSev samples were used to develop a MALDI-based N-glycan profiling method. The goal was to have a workflow that required minimal sample processing and could be completed in a 6–8 h timeline, in contrast to current glycomic analysis workflows for urine and prostatic fluids that require multiple processing, derivatization and purification steps prior to analysis. A previous slide-based approach used for serum and plasma N-glycan profiling (Blaschke et al., 2020) was the starting point, and a workflow summarized in Figure 1 was developed for EPSu. A key feature is use of an amine reactive slide chemistry that covalently binds target glycoproteins, facilitating washing steps to remove lipid and salts prior to spraying of a molecular coating of PNGase F PRIME to release N-glycans. An additional concentration and buffer exchange step was added for EPSu and EPSd, using a 10,000 MW cut-off spin cartridge to concentrate and allow buffer exchange of the sample prior to addition to the amine-reactive slide. This step results in a three to four fold increase in concentration of glycoproteins in the biofluid. An SDS-polyacrylamide gel image showing protein loading examples for the EPSu, EPSd and EPSev samples are provided in Supplementary Figure S1.

**FIGURE 2:**
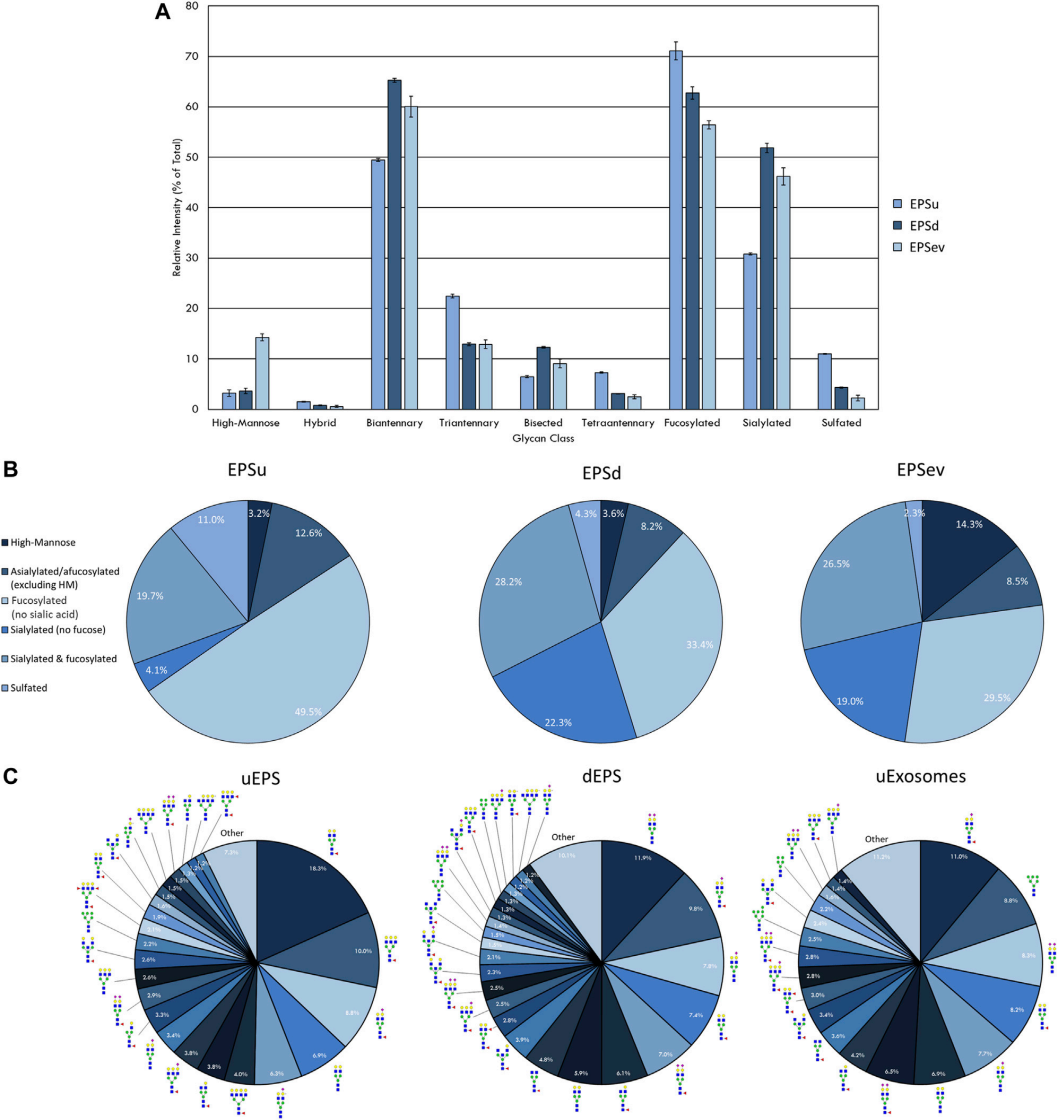
N-glycan profile of prostatic fluids (EPSu, EPSd, and EPSev) analyzed by MALDI-IMS. **(A)** Glycan class abundances of representative samples determined by summing the relative intensities of the N-glycans assigned to that class. Samples were analyzed in technical triplicate. Error bars represent the standard deviation. **(B)** Distributions of representative samples’ N-glycans assigned into groups based on composition. **(C)** Distribution of total N-glycan intensity by individual N-glycan, with N-glycans comprising at least 1% of the total intensity displayed and annotated. The N-glycan compositions are represented by blue squares for *N*-acetylglucosamine, green circles for mannose, yellow circles for galactose, purple diamonds for sialic acid, red triangles for fucose, and an “S” for sulfate.

Initial experiments focused on optimizing detection of N-glycans in EPSu and EPSd sample pairs obtained from the same donors, as well as existing EPSev pool samples. Similar to what was previously determined for serum or plasma preparation on the amine-reactive hydrogel slides, the key for optimal N-glycan detection was inclusion of the Carnoy’s solution wash after spotting, which serves to remove lipids and salts, as well as denature the bound glycoproteins facilitating access for PNGase F. Thus far, a total of 35 EPSu, 10 EPSd and 8 EPSev samples have been analyzed with the workflow shown in Figure 1. Cumulatively, the resulting N-glycans detected in each sample type are summarized in Supplementary Table S1, and structural class groupings are shown in Figure 2. Broadly, the EPSu samples had the most N-glycan species detected (*n* = 182) versus EPSd (*n* = 135). These numbers include multiple versions of the same N-glycan compositions for sialylated and sulfates species, which can vary in mass due to varying numbers of sodium ions associating with the charged groups. These glycoforms were included in Supplementary Table S1, but were generally detected at lower intensity values.

There was a range of protein concentrations across the samples in each sample type examined. While this created differences in the total intensity of the N-glycan profile and number of N-glycans that could be detected, these differences were accounted for by only comparing relative intensities, i.e. an individual N-glycan’s intensity relative to the total intensity of the N-glycans in that sample that were also seen in all sample types being compared. Representative samples with the most N-glycans detected were selected and compared from each sample type to display the breadth the N-glycan profiles.

The intensity of the N-glycan classes varied across the prostatic fluid samples (Figure 2A). EPSd and EPSev N-glycan classes had similar intensities, except for the higher amount of high-mannose N-glycans in EPSev. For all samples, the majority of the N-glycans were biantennary and/or fucosylated. EPSu had approximately 15 and 20% less sialylation than the EPSd and EPSev, respectively, but had an increased amount of tetraantennary and sulfated N-glycans. Many of these findings were replicated when grouping the N-glycans detected in each sample based on composition (Figure 2B). About half of the N-glycans in EPSu were fucosylated with no sialic acid compared to approximately 30% in the EPSd and EPSev samples. The EPSd and EPSev samples had higher levels of N-glycans with sialic acids and no fucose. When examining the intensity of individual N-glycans in the prostatic fluid samples, the most abundant N-glycans were typically biantennary with two galactoses (Figure 2C). In concordance with the N-glycan class comparison, EPSev had more high abundance high-mannose N-glycans than the other samples, and m/z 1419.4755 (Hex6HexNAc2 + 1Na) was the second most abundant N-glycan. The sulfated N-glycan m/z 2056.6156 (Hex5HexNAc4NeuAc1 + 1SO4 + 2Na) had a relative intensity of 6.3% in the EPSu, compared to 1.2% in EPSd and less than 1% in EPSev.

**FIGURE 3:**
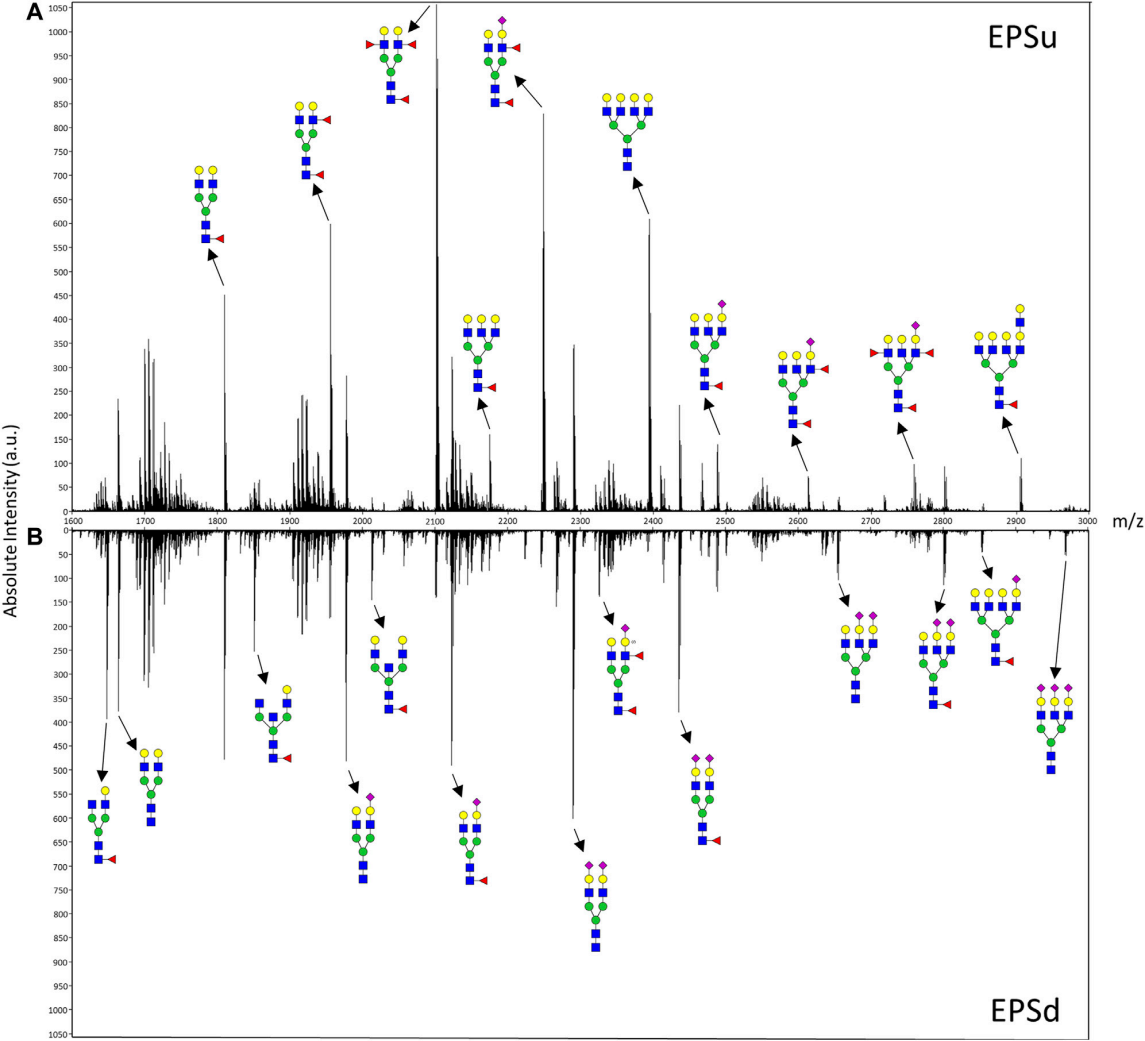
Mass spectra of representative EPSu **(A)** and EPSd **(B)** samples with annotations for a set of putative N-glycan structures. The N-glycan compositions are represented by blue squares for *N*-acetylglucosamine, green circles for mannose, yellow circles for galactose, purple diamonds for sialic acid, red triangles for fucose, and an “S” for sulfate.

In order to compare mass spectra and absolute intensity values of N-glycan peaks, an EPSu and EPSd sample with similar protein levels from the same cohort were prepped together and imaged in the same run (Figure 3). Substantial differences were seen in most of the high-intensity N-glycan peaks, except for m/z 1809.6393 (Hex5dHex1HexNAc4 + 1Na), m/z 2465.8669 (Hex6dHex1HexNAc5NeuAc1 + 1Na), and m/z 2800.9263 (Hex6dHex1HexNAc5NeuAc2 + 3Na). The increased levels of sialylated N-glycans in the EPSd sample and increased fucosylated species in the EPSu sample is also evident.

**FIGURE 4:**
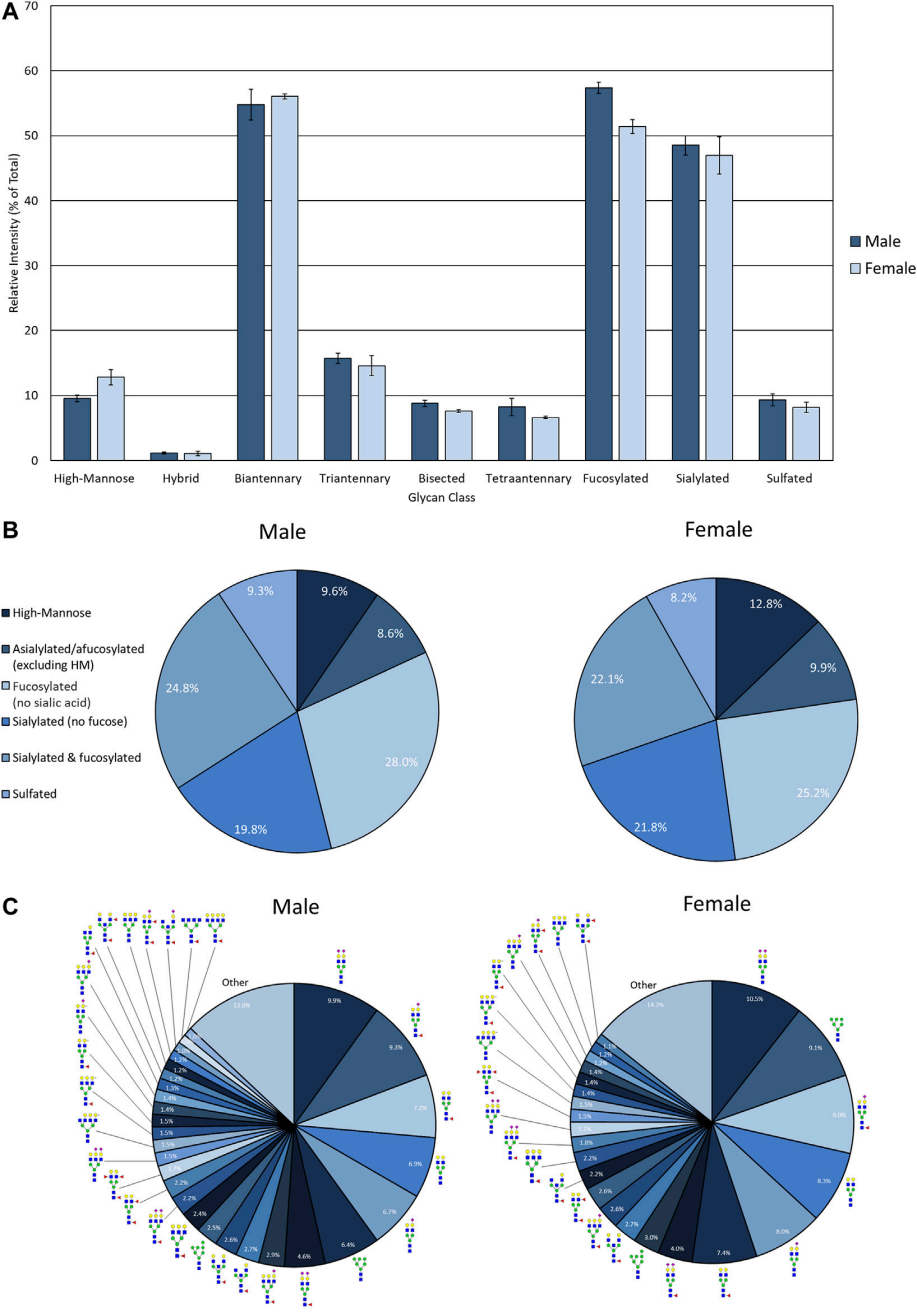
N-glycan profile of pooled healthy male and female urine analyzed my MALDI-IMS. **(A)** Glycan class abundances of representative samples determined by summing the relative intensities of the N-glycans assigned to that class. Sample were analyzed in technical quadruplicate. Error bars represent the standard deviation. **(B)** Distributions of representative samples’ N-glycans assigned into groups based on composition. **(C)** Distribution of total glycan intensity by individual N-glycan, with N-glycans comprising at least 1% of the total intensity displayed and annotated. The N-glycan compositions are represented by blue squares for *N*-acetylglucosamine, green circles for mannose, yellow circles for galactose, purple diamonds for sialic acid, red triangles for fucose, and an “S” for sulfate.

The advantage of developing the assay using EPSu samples are the inherently higher protein concentrations present from the prostatic fluid mixture in these samples relative to normal urine. Therefore, for comparison, control urine samples from pools of four healthy male and four healthy female donors were processed and analyzed using this workflow. This required 4 ml of starting fluid for concentration and desalting using larger filtration tubes, but was otherwise the same workflow as described for EPSu/d samples. Overall, N-glycan class intensities were similar for both samples (Figure 4A). There were high levels of biantennary, fucosylated, and sialylated N-glycans and low levels of hybrid N-glycans. The rest of the N-glycan classes had approximately 10% intensity. The biggest difference between the male and female samples was the slightly lower level of fucosylation in the female sample. The distribution of N-glycans based on composition displayed very similar profiles (Figure 4B). The biggest difference was a 3.2% increase in high-mannose N-glycans in the female sample. Accordingly, m/z 1419.4755 (Hex6HexNAc2 + 1Na) was the sixth most abundant N-glycan in males and the second most abundant in females (Figure 4C). Similar to the EPSu samples, the most abundant N-glycans are primarily biantennary with two galactoses.

**FIGURE 5:**
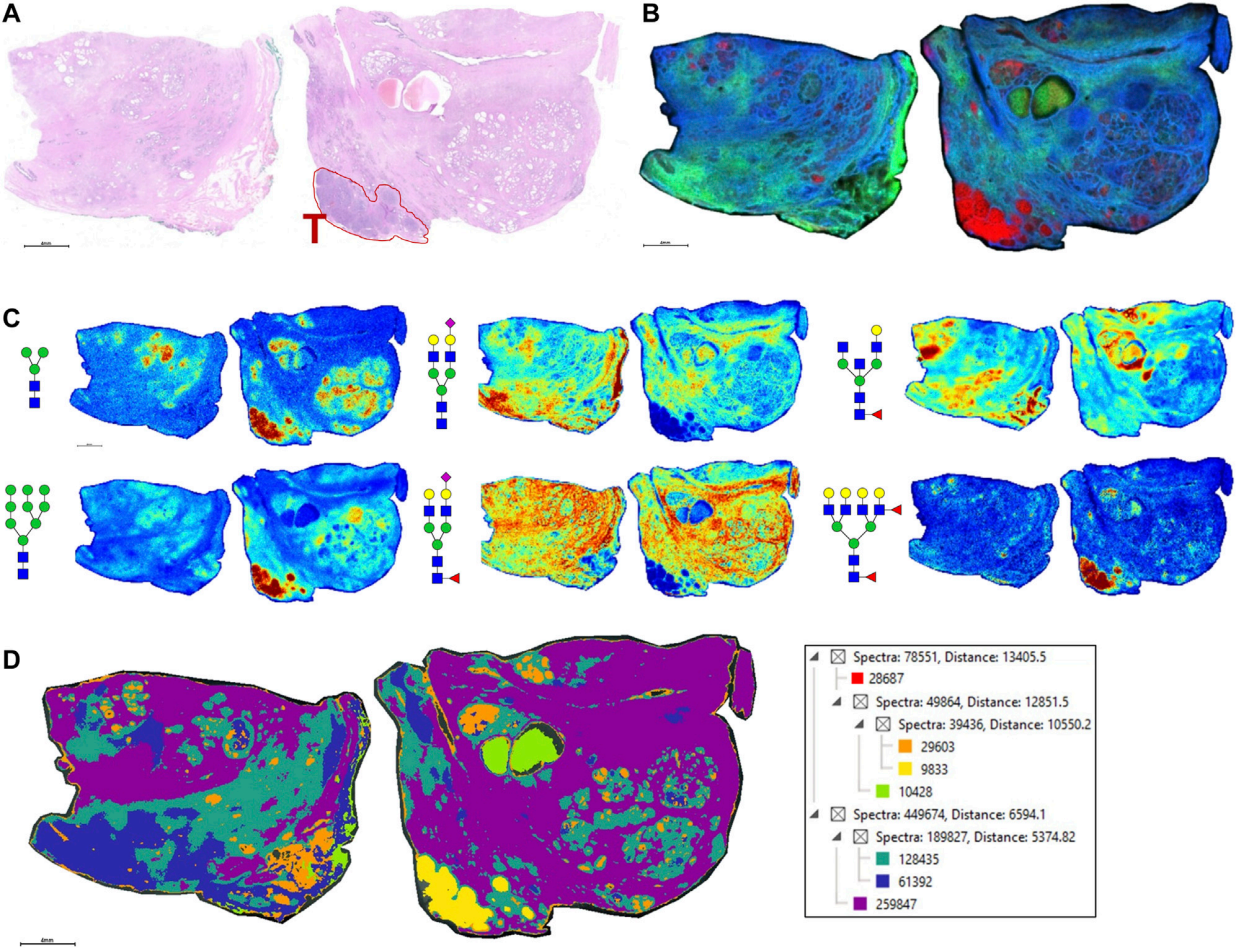
N-glycan imaging of FFPE prostate cancer and non-cancer tissues. **(A)** H&E stain of the two tissues from the same donor, non-cancer tissue on the left side and tumor on the right side. The tumor region, a Gleason grade 8/stage p3Tb, is highlighted with red outline and a red T. **(B)** An overlay MALDI image of three N-glycans, two stroma-associated ones, Hex5HexNAc4NeuAc1 in blue and Hex5HexNAc4Fuc1NeuAc1, and a tumor glycan Hex7HexNAc6Fuc2 (in red). **(C)** Six representative individual N-glycan images representative of different structural classes are shown, and glycan structure: a paucimannose (Hex3HecNAc2), high mannose (Hex5-Hex9HexNAc2), sialylated biantennary no fucose (Hex5HexNAc4NeuAc1), sialylated biantennary with fucose (Hex5HexNAc4Fuc1NeuAc1), bisecting GlcNAc biantennary (Hex4HexNAc4Fuc1) and branched fucosylated species (Hex7HexNAc6Fuc2). **(D)** Segmentation analysis, using Manhattan and k-bisecting classifications, was applied to the 73 N-glycans detected in the tissues. Spectra groupings are shown in the adjacent data tree. The N-glycan compositions are represented by blue squares for *N*-acetylglucosamine, green circles for mannose, yellow circles for galactose, purple diamonds for sialic acid, red triangles for fucose, and an “S” for sulfate.

**FIGURE 6:**
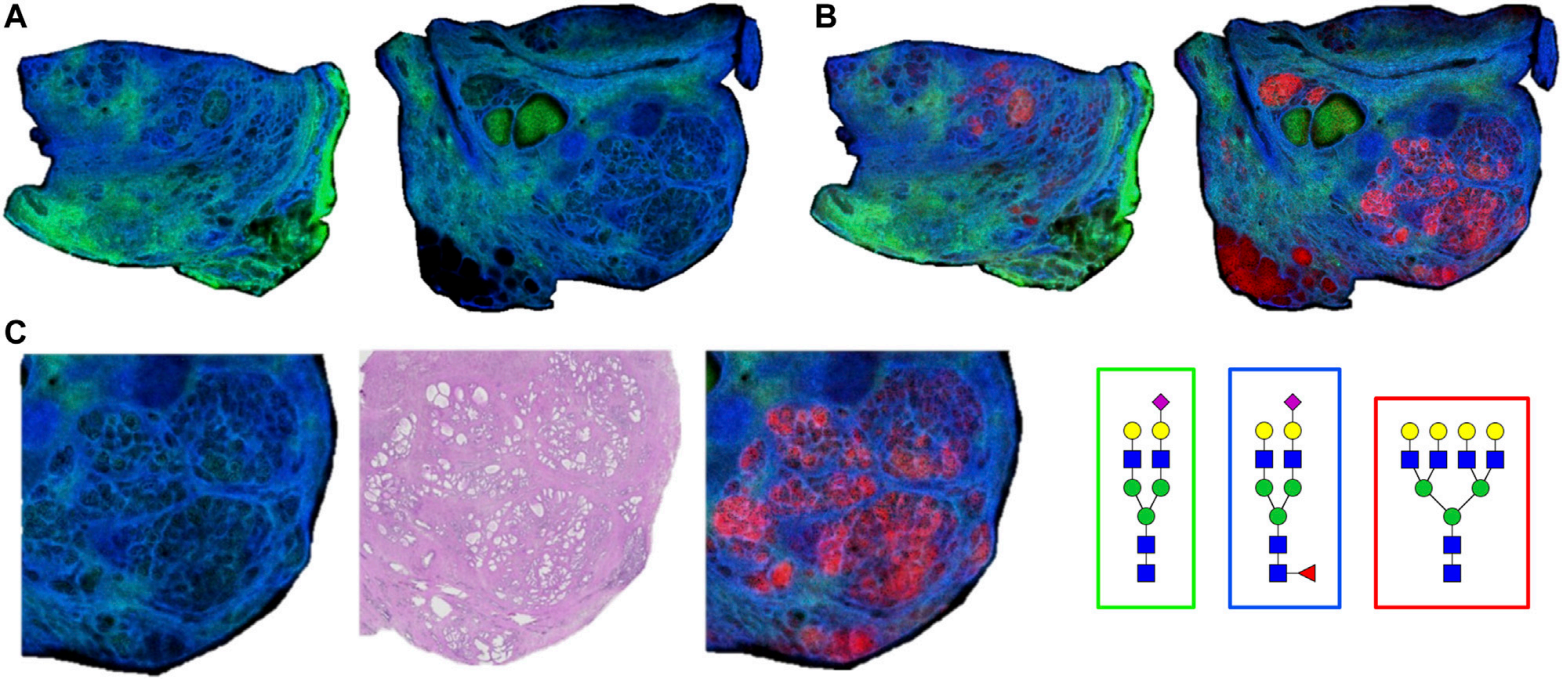
Detection of intralumenal glycans in non-tumor prostate glands. **(A)** Two distinct stroma-associated glycans, Hex5HexNAc4NeuAc1 in blue and Hex5HexNAc4Fuc1NeuAc1 in green, were chosen to highlight the locations of the prostate glands. **(B)** An example of a lumenal glycan is shown in red (Hex7HexNAc6), overlayed with the stroma glycans. **(C)** Highlighted region of the tumor tissue, bottom right corner, to illustrate the glycan distributions inside the glands. The analogous region in the H&E stained slide is also shown. Structures are bordered with the color that matches their tissue localizations.

As the glycoprotein constituents of the EPSu and EPSd samples are secreted by prostate glands, a comparative N-glycan comparison of prostate tissues was done using previously reported N-glycan MALDI IMS approaches (Drake et al., 2017, 2018b; McDowell et al., 2021). In the example shown in Figure 5A, a pair of non-tumor and tumor tissues from the same donor were evaluated. Use of the non-tumor tissue from the same donor is done to demonstrate the specificity of tumor specific N-glycans detected in the tumor containing tissue [a Gleason grade 8 (4 + 4), stage pT3b]. The tumor region is quite distinct, localized to the bottom left corner of the tissue. Additional H&E images of both tissues with increasing magnification are provided in Supplementary Figure S2. In Figures 5B,C are shown representative N-glycan classes and their tissue distributions, from a total of 73 N-glycans detected. These structures include tumor-associated paucimannose (Hex3HecNAc2), high mannose (Hex5-Hex9HexNAc2), and branched fucosylated species (Hex7HexNAc6Fuc2) (Figure 5C), consistent with previous reports (Drake et al., 2018b). Two of the most abundant sialylated biantennary N-glycans detected are stroma-associated, as shown in the overlay image with a tumor N-glycan (in red), Hex5HexNAc4NeuAc1 in blue and Hex5HexNAc4Fuc1NeuAc1 in green (Figure 5B, and individually in Figure 5C). A segmentation analysis of the 73 N-glycans is shown in Figure 5D, illustrating how different N-glycan classes are associated with different histopathology features. This representative tissue was selected for another feature, as it was noted that there was a distinct intra-lumen glandular N-glycan signature that could be detected. As shown in Figure 6A, the two sialylated biantennary N-glycans (Hex5HexNAc4NeuAc1 in blue and Hex5HexNAc4Fuc1NeuAc1 in green) provide a stromal scaffold image. This was used to detect which N-glycan species were present in the lumen regions, as illustrated in Figure 6B for Hex7HexNAc6 in red, and in Figure 6C for a highlighted gland region. Doing this, 38 N-glycans were detected in the lumen of glands. Many of these same N-glycans are also tumor associated, but the histopathology differences are significant between tumor and lumen.

**FIGURE 7:**
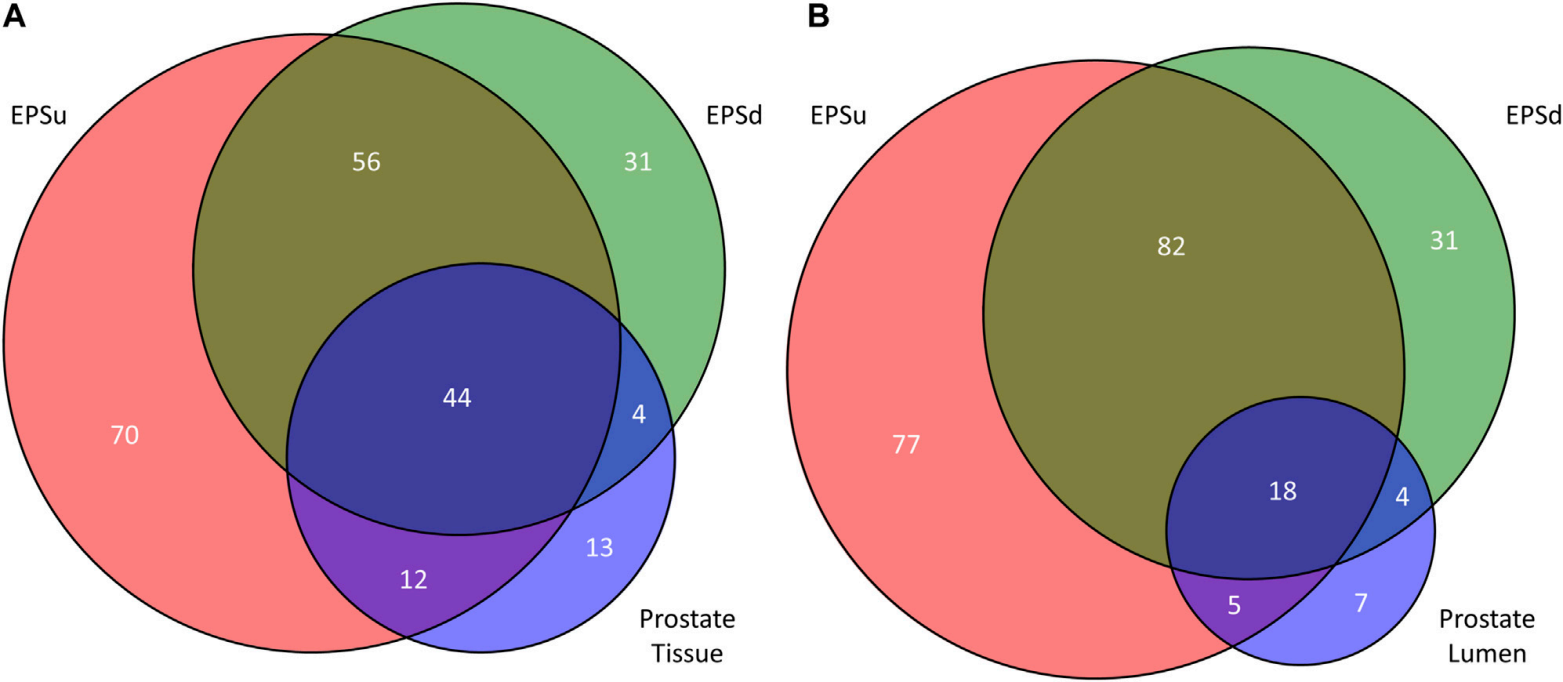
Comparison of detected N-glycans across sample sets. **(A)** The number of unique and common N-glycans detected in EPSu, EPSd, and across the entire prostate tissues. **(B)** The number of unique and common N-glycans detected in EPSu, EPSd, and within the lumen of the prostate tissues.

Using the lists of 73 tissue N-glycans and the subset of 38 lumen N-glycans, these were compared to the N-glycans detected in EPSu and EPSd samples. As shown in the Venn diagram in Figure 7, 44 N-glycans were present in the tissues and both EPS samples (Figure 7A), and 18 N-glycans were shared in the lumen and EPS samples (Figure 7B). Structurally, these shared N-glycans were the high mannose and the most abundant branched fucosylated N-glycans.

## Discussion

The goal of our study was to develop and optimize a more efficient and rapid method to evaluate N-linked glycosylation by MALDI-MS in urine and EPSu samples. The workflow is similar to what was developed for serum and plasma (Blaschke et al., 2020), utilizing an amine reactive slide to capture glycoproteins on a solid surface. An additional 1 h step of sample processing by molecular weight filtration and centrifugation for urine was included in order to concentrate and solvent exchange. Spotting only 1 ul, the urine glycoprotein spots are sprayed with a molecular coating of PNGase F, followed by analysis on a MALDI-FTICR or MALDI-QTOF mass spectrometer. The method is applicable to urine, EPSu and EPSd samples. An EPSev prep was spotted directly onto the amine reactive slides without any pre-processing. Routinely, 100 or more N-glycans can be detected depending on the sample type.

The N-glycan profiling of the prostatic fluid samples displayed more high-mannose N-glycans in the EPSev compared to the EPSu and EPSd, reflected in the intensities of the N-glycan classes and the most abundant individual N-glycans (Figure 1). Relatively high intensities of high-mannose N-glycans have been noted in non-EPS urinary exosomes as well (Zou et al., 2017). The similarity of the EPSd and EPSev N-glycan profiles suggests that a significant portion of the EPS glycoproteins may be within exosomes. In conjunction with these findings, direct comparison of mass spectra from EPSu and EPSd with similar protein levels displayed distinct N-glycan profiles for EPSu and EPSd (Figure 2). While male and female urine N-glycan profiles and individual N-glycan abundances displayed few differences, there was a slightly higher amount of fucosylation in the male sample. This trend has previously been identified in male and female plasma samples (Dotz and Wuhrer, 2019). Similar levels of sulfation were detected in the urine controls and EPSu, which was higher than the EPSd and EPSev, indicating that the sulfated N-glycans in EPSu may be originating from urine glycoproteins.

A comprehensive MS analysis of the N-glycans present in adult and pediatric urine samples has been published (Li et al., 2020). In this study, PNGase F released N-glycans were isolated, labeled with aminobenzoic acid, and methylamidated prior to analysis by LC-MS/MS. The authors reported 116 N-glycan compositions could be detected, and a subset of 46 N-glycans that were reproducibly detected and further quantified (Li et al., 2020). This study provides an optimal benchmark for our current study to evaluate how many of the 46 N-glycans could be detected by our solid-phase and single day MALDI MS workflow. Comparing the MALDI data from the male and female urine standards, the majority of N-glycans are detected, especially the biantennary, high mannose and fucosylated species. The MALDI-based assay is not as effective for detection of larger tri- and tetra-sialylated N-glycans, and for N-glycans with molecular masses above 3,700 m/z.

One observation noted from our data was that the intensity levels of N-glycans detected were variable, even when protein concentrations were comparable. There was also not a large variation in the types of N-glycan structures detected in the samples, especially for the most abundant species. These variations could reflect significantly different protein levels of individual glycoprotein species, or variations in the amount of the most abundant protein in human urine, uromodulin. Uromodulin, also called Tamm−Horsfall protein, has eight N-glycosylation sites and has been comprehensively studied for N-glycosylation content and composition (Li et al., 2021). Interestingly in this study, N-glycan analysis comparisons of a uromodulin depleted urine versus non-depleted sample indicated little difference in the N-glycan compositions detected in both samples (Li et al., 2021). Our study only represents a few sample numbers, so no conclusions can be reached until statistically relevant sample numbers are analyzed. The rapidity and efficiency of the slide-based MALDI assay will facilitate evaluation of larger cohorts. Possibly pairing the N-glycan data with the quantitative MRM proteomic assay developed for EPSu proteins could address both the changes in N-glycan levels and protein concentrations in the same sample (Kim et al., 2016; Otto et al., 2020). It also does not preclude direct targeting of specific glycoproteins present for N-glycan content analysis, as is done so often for PSA. We have already reported a solid-phase antibody array approach to N-glycan profile individual serum glycoproteins captured by their specific antibodies (Black et al., 2019a; 2019b). A similar approach targeting the abundant urine and EV glycoproteins like PSA is in progress.

The N-glycans obtained from a representative FFPE prostate cancer tissue were also included to illustrate the N-glycans that can be detected in the lumen of the prostate glands, representing prostatic fluid still present during tissue fixation. Based on hundreds of prostate cancer tissues analyzed by N-glycan IMS, to be reported separately, detection of luminal N-glycans in these tissues is highly variable. Fluid remnants can be seen in the H&E stains, but not all gland lumens contain this, and can be completely absent in many tissues. Presumably this variation reflects how much fluid was present at the time of prostatectomy, but could also reflect differences in FFPE tissue preparation and processing. While this is a variable that may lack clinical diagnostic significance, the selected tissue in Figure 5 highlights the tumor-associated and secreted N-glycans (*n* = 38) in tissue that can potentially be detected in the EPSu, EPSd and EPSev samples. These are primarily paucimannose, high mannose and branched multi-fucosylated N-glycans.

In summary, we present an initial N-glycan profiling workflow applicable to urine and prostatic fluids. It also represents a scalable framework, depending on the application and intent of the assay. There is already demonstrable sensitivity for detecting N-glycans efficiently in low concentrations of starting material, hence analysis of larger clinical cohorts is feasible. If time of preparation is less of a concern, an increased amount of starting fluid can be concentrated to increase protein concentrations prior to spotting. Additionally, the use of the concentration and solvent/buffer exchange step in the workflow can be adapted to other biofluids with protein concentrations similar to urine, i.e., saliva, cerebrospinal fluid, bronchial lavage. The method can also be used with other enzymes besides PNGase F, like endoglycosidase F3 specific to core fucoses (West et al., 2020), or other glycosidases specific to other N-glycan structural classes.

## Data Availability

The raw data supporting the conclusion of this article will be made available by the authors, without undue reservation.
