# Supplementary material for: Direct N-Glycosylation Profiling of Urine and Prostatic Fluid Glycoproteins and Extracellular Vesicles
Source: Front Chem. 2021 Sep 27;9:734280. doi: 10.3389/fchem.2021.734280 (PMC8503230; doi:10.3389/fchem.2021.734280)
Supplement: Supplementary file 1 [file Presentation1.pdf]

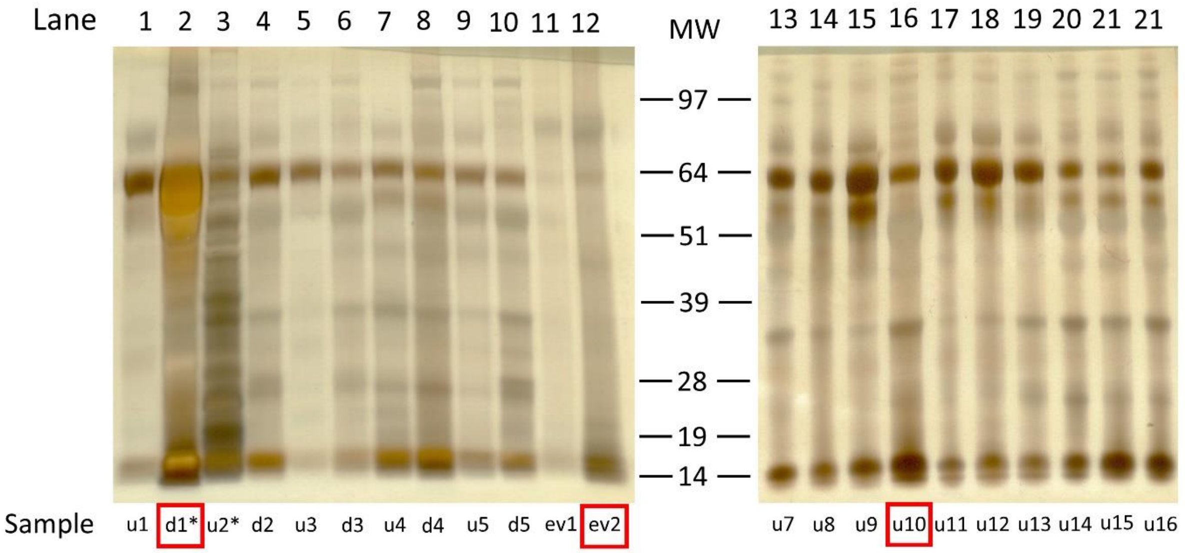

Supplementary Figure 1. SDS Gel separation of the sample preps for EPSu (labelled with a "u") and EPSd (labelled with a "d") sample pairs, and EPSev (labelled with "ev"). One microliter (approximately 10-15 ug protein) of each sample was added in 10 ul SDS loading buffer and separated on a 10% Genescript ExpressPlus polyacrylamide gel in MOPS buffer. SeeBlue Plus2 prestained molecular weight standards frpm Invitrogen were also loaded. Proteins were visualized by silver staining. Sample labels with an asterisk were used for mass spectra comparison, and sample labels with a red box were used as representative samples.

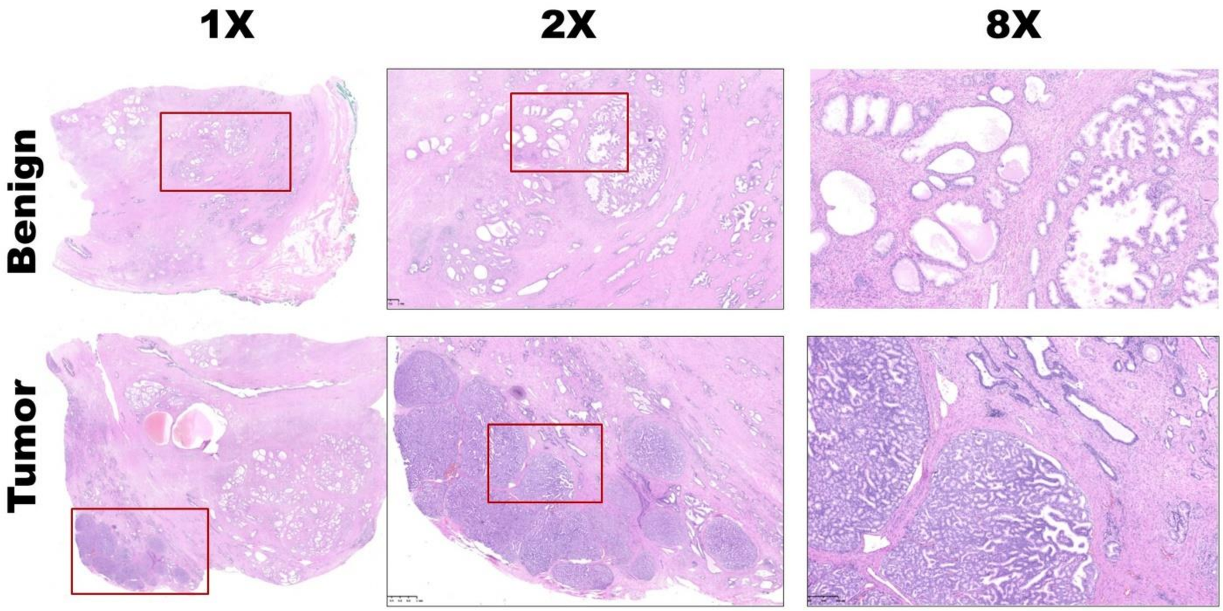

Supplementary Figure 2. Higher resolution images of the H&E stained prostate tissues. Images were obtained following H&E staining of each slide and scanning into a Hamamatsu Nanozoomer 2.0RS digital slide scanner. Images of 2X and 8X are shown for selected regions, highlighted in the preceding image with a red box outline.
